# Exercise Intervention in Autonomic Function, Immunity, and Cardiovascular Health: A Precision Medicine Approach

**DOI:** 10.3390/jcdd12070247

**Published:** 2025-06-26

**Authors:** Jianyu Li, Junbei Bai, Guochun Liu, Ziyan Zhu, Chunmei Cao

**Affiliations:** 1 Division of Sports Science and Physical Education, Tsinghua University, Beijing 100084, China; jy-li22@mails.tsinghua.edu.cn (J.L.); lgc890206@163.com (G.L.); 2 Competitive Sports No.4 Room, Beijing Research Institute of Sports Science, Beijing 100075, China; jujbeibai@hotmail.com; 3 College of Exercise Medicine, Chongqing Medical University, Chongqing 400331, China; 4 School of Pharmaceutical Sciences, Tsinghua University, Beijing 100084, China; zhuzy22@mails.tsinghua.edu.cn

**Keywords:** precision medicine, autonomic nervous system, immune system, cardiovascular health, physical activity

## Abstract

The imbalance in the interaction between the autonomic nervous system and the immune system serves as a central mechanism in the onset and progression of cardiovascular diseases. The excessive activation of the sympathetic nervous system and suppression of vagal function contribute to chronic inflammation and cardiac remodeling. Precision medicine, by integrating multidimensional data such as genomics and metabolomics, offers a novel perspective for the personalized design of exercise interventions. This systematic review explores the bidirectional regulatory mechanisms of exercise interventions on the autonomic nervous system–immune axis and examines the potential applications of precision medicine in optimizing exercise prescriptions and clinical translation. Exercise significantly improves cardiovascular function through immunometabolic reprogramming, which includes suppressing sympathetic overactivity, enhancing vagal tone, and modulating the IL-6/IL-10 balance, as well as activating the short-chain fatty acid (SCFA)–Treg axis. Moreover, precision-medicine-driven ACE I/D gene typing provides a basis for selecting tailored exercise prescriptions, thereby significantly enhancing the efficacy of exercise interventions. By leveraging a multi-tiered “neuro–immune–metabolic” regulatory framework, exercise interventions contribute to improved cardiovascular health. The application of precision medicine technology overcomes individual variability constraints, advancing exercise prescription design from generalized recommendations toward personalized and dynamically adaptive strategies.

## 1. Introduction

The autonomic nervous system (ANS) precisely regulates cardiovascular function through the dynamic balance between the sympathetic and vagal nerves. Increased sympathetic excitability accelerates the heart rate and induces vasoconstriction, temporarily enhancing the cardiac output, whereas the vagus nerve counterbalances this effect by releasing acetylcholine to slow the heart rate and dilate blood vessels, thereby maintaining the body’s resting state [1]. Heart rate variability (HRV) serves as a key indicator for assessing autonomic nervous function, reflecting the dynamic interplay between sympathetic and vagal activity. Clinical studies have demonstrated that reduced HRV—indicating sympathetic dominance or vagal inhibition—is strongly associated with the incidence, clinical prognosis, and mortality of various cardiovascular diseases, including coronary artery disease and heart failure. The underlying mechanisms involve chronic inflammation, oxidative stress, and myocardial remodeling, driven by excessive sympathetic activation through β-adrenergic-receptor-mediated NF-κB pathway activation and the subsequent pro-inflammatory cytokine overproduction [2,3].

Exercise interventions hold great potential in cardiovascular health management, particularly in regulating the ANS and immune system. Regular physical activity improves autonomic function by promoting the balance between sympathetic and parasympathetic activity, effectively reducing the risk of cardiovascular diseases [4]. Additionally, exercise enhances immune tolerance and exerts anti-inflammatory effects, further optimizing cardiovascular health and lowering the risk of atherosclerosis and other related conditions [5]. While moderate exercise demonstrates cardioprotective effects, excessive physical activity may disrupt the equilibrium between reactive oxygen species (ROS) production and antioxidant defenses, potentially triggering cellular damage and maladaptive immune responses [6]. However, the primary limitations of traditional exercise prescriptions lie not only in their neglect of individual variability, but also in their static and experience-based approach to dose design. The inherent ambiguity in exercise dosage—such as the broad and imprecise definition of “moderate intensity”—results in highly heterogeneous intervention outcomes. This stands in stark contrast to the precise dose–response control commonly achieved in pharmacological treatments [7]. Therefore, to transcend the constraints of conventional frameworks, it is essential that we reconstruct the design paradigm of exercise prescriptions from the perspective of “precision dosing”. For instance, angiotensin-converting enzyme (ACE) gene insertion/deletion (I/D) polymorphisms influence Ang-II levels, thereby altering an individual’s sympathetic inhibition threshold in response to high-intensity interval training. This limitation has driven the integration of precision medicine into exercise prescription design, allowing for more tailored and effective interventions [8].

Precision medicine is a personalized healthcare approach that tailors medical interventions based on an individual’s genetic, metabolic, and environmental characteristics. By integrating genomic, metabolomic, and phenotypic data, precision medicine enables the analysis of inter-individual differences in response to exercise interventions, thereby optimizing personalized exercise prescriptions to enhance cardiovascular health outcomes [9]. For instance, the alpha-actinin-3 (ACTN3) gene polymorphism R577X has been identified as a predictor of muscle adaptability [10], while the peroxisome-proliferator-activated receptor gamma coactivator 1-alpha (PPARGC1A) gene polymorphism rs8192678 (Gly482Ser) is directly linked to mitochondrial biogenesis efficiency [11], Moreover, metabolomic studies have revealed that exercise-induced changes in short-chain fatty acids (SCFAs) can modulate T-cell differentiation, thereby balancing immune–inflammatory responses [12]. This review systematically explores, from a precision medicine perspective, the physiological mechanisms by which exercise interventions improve cardiovascular health through the “autonomic–immune–metabolic” axis. Additionally, it examines how multi-omics technologies facilitate the transition of exercise prescriptions from generalized recommendations to personalized, dynamically regulated strategies, offering novel approaches for the prevention and rehabilitation of cardiovascular diseases.

## 2. Materials and Methods

To conduct this review, we searched the PubMed, EBSCO, Embase, Scopus, and Web of Science databases using predefined search terms for English-language articles published between 1 January 2015 and 31 February 2025 encompassing three domains: (1) exercise interventions: exercise, physical activity, physical exercise, acute exercise, isometric exercise, aerobic exercise, and high-intensity interval training; (2) autonomic–immune interactions: autonomic nervous system, neuro–immune axis, cytokines, heart rate variability, and cardiac remodeling; and (3) precision medicine: genomics, metabolomics, and personalized exercise prescription. Both controlled vocabulary terms and free-text keywords were applied, with Boolean operators AND/OR used to refine search combinations. This review follows the preferred reporting items for systematic reviews and meta-analysis guidelines. Two researchers independently performed the literature search and screening. In cases of disagreement, further discussion was conducted to reach a consensus. Data extraction from the selected studies was also performed independently by both researchers.

**Figure 1:**
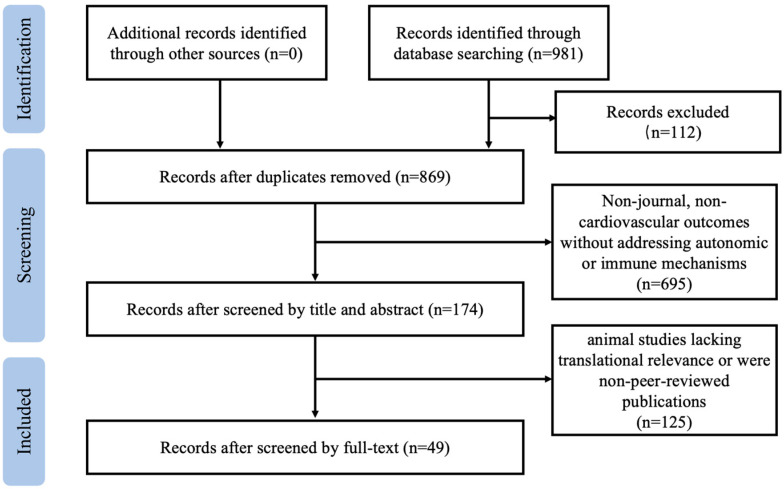
Flowchart of literature search and inclusion/exclusion process.

Eligible studies included original research articles, randomized controlled trials, prospective cohort studies, meta-analyses, and systematic reviews published in English that investigated the mechanistic role of exercise in autonomic–immune regulation or integrated precision medicine approaches toward exercise prescription design. Studies were excluded as follows: (1) they focused on non-cardiovascular outcomes without addressing autonomic or immune mechanisms; or (2) they were animal studies lacking translational relevance or were non-peer-reviewed publications. Two independent reviewers screened titles, abstracts, and full texts for eligibility, resolving discrepancies through discussion or consultation with a third reviewer. Specific literature inclusion and exclusion processes are shown in Figure 1.

## 3. Autonomic–Immune Interactions: Theoretical Mechanisms and Exercise Modulation

### 3.1. Regulatory Pathways of the ANS in Immune Modulation

**Figure 2:**
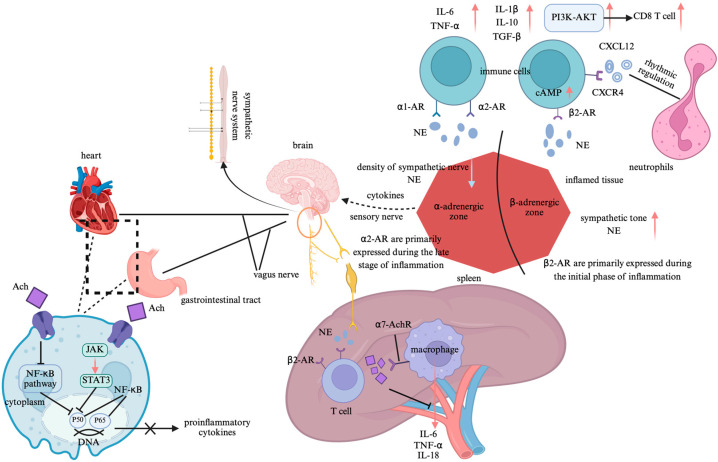
Neuroimmune regulatory pathways of the autonomic nervous system. Note: Created in https://BioRender.com; 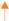: promote; 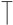: inhibit. Abbreviation: NF-κB: nuclear factor-kappa B; JAK-STAT3: janus kinase 2-signal transducer and activator of transcription 3; Ach: acetylcholine; β2-AR: β2-adrenergic receptors; NE: norepinephrine; α7-AchR: α7 nicotinic acetylcholine receptors; IL-1β: interleukin-1β; IL-6: interleukin-6; IL-10: interleukin-10; IL-18: interleukin-18; TNF-α: tumor necrosis factor-alpha; TGF-β: transforming growth factor-beta; cAMP: cyclic adenosine monophosphate; α1-AR: α1-adrenergic receptors; α2-AR: α2-adrenergic receptors; PI3K-AKT: phosphatidylinositol-3-kinase/protein kinase B; CD8 T cell: Cytotoxic T lymphocyte; CXCL12: Chemokine (C-X-C motif) ligand 12; CXCR4: Chemokine (C-X-C motif) receptor type 4.

The ANS dynamically regulates immune homeostasis through the synergistic and antagonistic actions of the sympathetic and vagal nerves. This regulatory mechanism involves molecular interactions between neurotransmitters and receptors, central–peripheral signal integration, and the remodeling of the immune organ microenvironment (Figure 2) [13,14,15].

The interaction between neurotransmitters and immune cell receptors constitutes the fundamental mechanism by which the ANS modulates immune function. The sympathetic nervous system (SNS) non-specifically releases norepinephrine (NE) from its nerve terminals, which binds to β_2_-adrenergic receptors (β2-ARs) to promote the production of anti-inflammatory cytokines such as interleukin-10 (IL-10) and transforming growth factor-beta (TGF-β), exhibiting a pronounced anti-inflammatory effect [13]. In contrast, NE binding to α_1_- and α_2_-adrenergic receptors enhances the secretion of pro-inflammatory cytokines such as tumor necrosis factor-alpha (TNF-α), thereby exerting a pro-inflammatory effect [16]. Additionally, NE activates the phosphatidylinositol-3-kinase/protein kinase B (PI3K/Akt) pathway, driving the migration of CD8^+^ T cells and significantly enhancing their mobility. Moreover, NE- regulates neutrophil rhythmic release via the CXCL12-CXCR4 axis [17]. Experimental studies further underscore the critical role of neurotransmitter–receptor molecular interactions in immune balance regulation. In adrenergic receptor β2 gene knockout (Adrb2^−^/^−^) mice or in mice treated with the β2-AR antagonist zenidolol, granulocyte–monocyte progenitor (GMP) proliferation in the spleen was significantly suppressed. Conversely, in sympathectomy-induced (6-OHDA-treated) mice, the morning peak release of neutrophils was markedly reduced, confirming the essential role of sympathetic neurotransmission in immune regulation [18,19]. The parasympathetic nervous system (PNS) primarily modulates immune function through the cholinergic anti-inflammatory pathway. This pathway facilitates the release of acetylcholine (Ach), which binds to and activates α7 nicotinic acetylcholine receptors (α7-AchR). This activation inhibits janus kinase 2-signal transducer and activator of transcription 3 (JAK2-STAT3) signaling, which directly suppresses nuclear factor-kappa B (NF-κB) nuclear translocation through the STAT3-dependent phosphorylation of inhibitor of NF-κB alpha, leading to the cytoplasmic retention of NF-κB. This blockade inhibits TNF-α transcription and enhances anti-inflammatory responses [20]. As the largest immune organ, the spleen plays a pivotal role in the cholinergic anti-inflammatory pathway. The anatomical structure of the spleen comprises two key neuronal components: the preganglionic neurons, which originate from the dorsal motor nucleus of the vagus nerve and terminate at the celiac–superior mesenteric ganglia, and the postganglionic adrenergic neurons, which extend via the splanchnic nerve to the spleen, surrounding immune cells. In splenic denervation models, vagal nerve stimulation fails to suppress TNF-α production, demonstrating that adrenergic neurons within the splenic nerve are critical for inflammatory inhibition [21]. Furthermore, in β2-AR knockout mice with sepsis, excessive TNF-α production has been observed. Treatment with a β2-AR agonist significantly alleviates the inflammatory response, indicating that β2-AR on immune cells participates in the cholinergic anti-inflammatory pathway, with its effects being dependent on Ach and α7-AchR [22].

The integration of multilevel signaling between the central nervous system (CNS) and immune organs is a crucial mechanism by which the ANS modulates immune function. The sympathetic central regulation of immunity involves the paraventricular nucleus (PVN) of the hypothalamus, which detects peripheral interleukin-6 (IL-6) signals and subsequently activates sympathetic premotor neurons in the rostral ventrolateral medulla (RVLM). This activation significantly increases the splenic sympathetic nerve discharge, thereby enhancing immune modulation. Additionally, microinjections of interleukin-1β (IL-1β) into the PVN have been shown to increase renal sympathetic nerve activity, a response dependent on the prostaglandin E2 receptor EP3 subtype (PGE2-EP3) signaling pathway [23,24]. Furthermore, electrical stimulation of the aortic depressor nerve, which activates the baroreflex, significantly reduces local neutrophil migration in the knee joint and lowers the secretion levels of TNF-α, IL-1β, and IL-6. This immunosuppressive effect is mediated by inhibiting the RVLM sympathetic output, thereby alleviating local joint inflammation. Notably, this effect disappears following a sympathetic chain transection, highlighting the central role of sympathetic inhibition in immune regulation [25]. In contrast, the cholinergic circuit of the PNS modulates immune function through a complex “vagal–sympathetic–immune” three-tiered feedback loop. Specifically, the celiac ganglion activates the splenic sympathetic nerve, leading to enhanced NE release, which, in turn, promotes Ach secretion in memory T cells. In septic mouse models, this pathway significantly suppresses TNF-α expression, demonstrating its anti-inflammatory potential [26]. Additionally, afferent fibers of the vagus nerve can detect pathogens through NOD-like receptors (NLRs) and activate the nucleus tractus solitarius (NTS)–dorsal motor nucleus of the vagus nerve (DMV) pathway. This activation inhibits toll-like receptor 4 (TLR4) signaling in intestinal macrophages, further underscoring the vagal nerve’s role in immune homeostasis.

Microenvironment-dependent immune remodeling exhibits significant organ specificity, determined by the local receptor distribution and immune cell differentiation status. In the spleen, the β2-AR signal inhibits B-cell IgM secretion, enhances interleukin-21 (IL-21) expression by follicular helper T cells (Tfh), promotes germinal center formation, and augments CD8+ T-cell function, thereby facilitating cell-mediated immunity. In contrast, the parasympathetic signal, through α7-AchR activation, suppresses ROS level in red pulp macrophage, reducing erythrophagocytosis [27]. Within the gut microenvironment, intrinsic layer sympathetic neurons inhibit type 2 innate lymphoid cell (ILC2)-mediated interleukin-5 (IL-5) secretion via α2-AR, thereby attenuating anti-helminth immunity. Meanwhile, parasympathetic vagal signaling activates cholinergic neurons in the enteric myenteric plexus, which, through muscarinic M3 receptors, suppresses the interferon-gamma (IFN-γ) expression in intraepithelial lymphocytes (IELs), further shaping local immune responses [28]. In joint tissues, SNS activity regulates chemokine secretion and neutrophil migration. Specifically, the β2-AR signal reduces CXCL1 secretion, while baroreflex activation lowers the local NE level, temporarily lifting the suppression on CXCL1. However, the net effect remains a reduction in neutrophil migration, possibly due to the sympathetic modulation of vascular permeability or direct immune cell interactions [29]. Moreover, the autonomic regulation of the joint immune microenvironment is highly specific. The knee joint is exclusively modulated by sympathetic nerves (L2–L6 segments), and the surgical transection of the lumbar sympathetic chain completely abolishes the baroreflex-mediated anti-inflammatory effects on the severed side, whereas the effect remains intact on the contralateral side. This highlights the crucial role of localized sympathetic pathways in joint-specific immune regulation [25].

### 3.2. Feedback Regulation of the ANS by the Immune System

**Figure 3:**
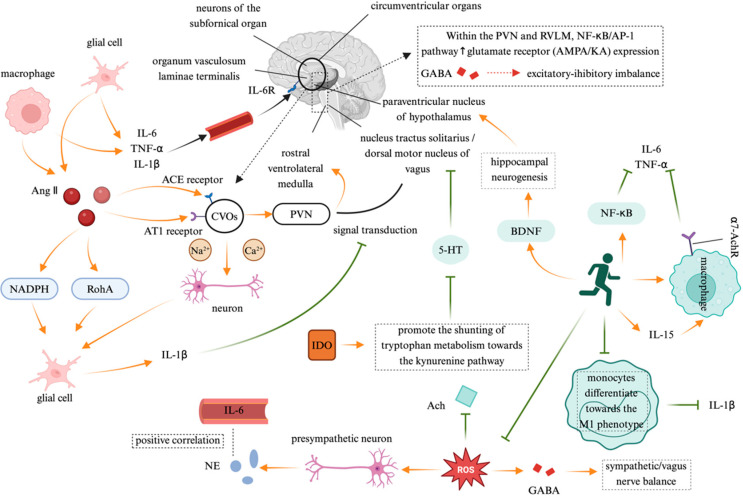
The feedback regulation of the autonomic nervous system by the immune system. Note: Created in https://BioRender.com; 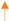: promote; 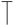: inhibit. Abbreviation: Ang II: angiotensin II; NADPH: nicotinamide adenine dinucleotide phosphate; RhoA: Ras homolog gene family member A; AT1: angiotensin type 1; CVOs: circumventricular organs; PVN: paraventricular nucleus; RVLM: rostral ventrolateral medulla; GABA: gamma-aminobutyric acid; NF-κB/AP-1: nuclear translocation of nuclear factor-kappa B/adaptor protein 1; 5-HT: 5-hydroxytryptamine; BDNF: brain-derived neurotrophic factor; ROS: reactive oxygen species; IL-6: interleukin-6; TNF-α: tumor necrosis factor-alpha; IL-1β: interleukin-1β; ACE receptor: Angiotensin-converting enzyme receptor; NE: norepinephrine; Ach: Acetylcholine; IDO: indoleamine 2,3-dioxygenase; α-7AchR: alpha-7 acetylcholine receptor.

The interaction between the immune system and the ANS is bidirectional, with immune-derived bioactive substances exerting feedback regulation on autonomic function. This feedback loop is particularly pronounced in chronic inflammatory diseases (Figure 3).

Proinflammatory cytokines directly influence the SNS, creating a self-reinforcing “inflammation–sympathetic hyperactivity” cycle. Anatomical studies have identified circumventricular organs (CVOs)—brain regions lacking a complete blood–brain barrier (BBB)—as key neuroimmune interfaces that facilitate the passage of peripheral IL-6, TNF-α, and IL-1β, which act on the CNS via macrophage and glial cell signaling. Experimental studies have demonstrated that peripheral IL-1β injection activates cytokine receptors in the vagus nerve, suppressing neural transmission from the NTS to the hypothalamus. This results in an enhanced sympathetic activity and a reduced vagal tone, leading to a significant increase in the action potential firing frequency of subfornical organ (SFO) neurons. The excitatory glutamatergic synaptic transmission from the SFO to the PVN and the RVLM further activates the sympathetic premotor neurons [30]. In cardiovascular disease (CVD) patients, plasma IL-6 levels are positively correlated with norepinephrine NE markers of SNS activity, suggesting that proinflammatory cytokines may stimulate RVLM sympathetic premotor neurons, exacerbating sympathetic overactivation [31]. Excessive CNS activation serves as a central hub linking immune responses to autonomic regulation. Angiotensin II (Ang-II) and inflammatory cytokines coactivate CVO neurons, directly increasing sodium/calcium currents and triggering early sympathetic excitation via a “rapid pathway”. Ang-II binding to angiotensin type 1 receptors (AT1R) in astrocytes and microglia induces oxidative stress and inflammatory cytokine release, further upregulating the brain angiotensin-converting enzyme (ACE) and AT1R expression, thereby reinforcing a positive feedback loop [32]. In the PVN and RVLM, inflammation enhances glutamate receptor (AMPA/KA) expression via the NF-κB/adaptor protein 1 (AP-1) signaling pathway, while inhibiting gamma-aminobutyric acid (GABA)ergic synaptic transmission, shifting the excitatory–inhibitory balance toward sympathetic excitation [33]. M1-polarized microglia play a central role in this process. Ang-II activates nicotinamide adenine dinucleotide phosphate (NADPH) oxidase and the Ras homolog gene family member A (RhoA)/Rho-associated coiled-coil forming protein kinase (ROCK) pathway, prompting the microglia to release IL-1β, which, in turn, triggers inflammasome activation via P2X7 receptor-dependent mechanisms. This “neuron–glia–immune” triadic interaction ultimately sustains sympathetic hyperactivity and accelerates cardiac remodeling [34]. Moreover, the immune system also indirectly remodels ANS function by modulating the neurotransmitter metabolism. Proinflammatory cytokines such as IFN-γ and TNF-α activate indoleamine 2,3-dioxygenase (IDO), shifting the tryptophan metabolism toward the kynurenine pathway, which suppresses serotonin synthesis while increasing neurotoxic metabolites such as quinolinic acid [35]. Reduced serotonin synthesis exacerbates depressive symptoms and, via 5-HT1A receptor signaling, suppresses the excitatory output from the dorsal motor nucleus of the vagus nerve (DMV), leading to a diminished vagal tone. Additionally, inflammation-induced oxidative stress disrupts mitochondrial function in cholinergic neurons, impairing acetylcholine synthesis, thereby exacerbating the autonomic imbalance characterized by sympathetic predominance and vagal suppression [36].

Exercise serves as an effective modulator of immune-driven autonomic dysfunction, exerting multi-faceted neuroimmune regulatory effects. Regular aerobic exercise suppresses TLR4 signaling in monocytes by downregulating ROS-dependent myeloid differentiation primary response 88/TIR domain-containing adaptor-inducing interferon-β adaptor protein recruitment, which attenuates IκB kinase phosphorylation and the subsequent NF-κB nuclear translocation, ultimately reducing the transcriptional activation of proinflammatory cytokines including IL-6 and TNF-α. Additionally, exercise-induced cytokines such as interleukin-15 (IL-15) enhance the cholinergic anti-inflammatory pathway, upregulating the α7-AchR-mediated inflammation suppression. Furthermore, regular exercise reduces oxidative stress in the RVLM, restores GABAergic inhibitory synaptic function, and improves the sympathetic–vagal balance [37]. The underlying mechanisms involve inhibiting the differentiation of peripheral monocytes/macrophages into the proinflammatory M1 phenotype, thereby lowering the IL-1β levels. Exercise also downregulates CNS activity, disrupts the Ang-II/AT1 signaling axis, and enhances BBB integrity, thereby preventing the infiltration of peripheral inflammatory mediators into the CNS [38]. Moreover, exercise contributes to the upregulation of the brain-derived neurotrophic factor (BDNF), promoting hippocampal neurogenesis and restoring the hypothalamus–NTS–vagus nerve feedback pathway, thereby reinforcing the homeostatic regulation of autonomic function [39].

### 3.3. The Bidirectional Regulatory Effects of Exercise Intervention

**Figure 4:**
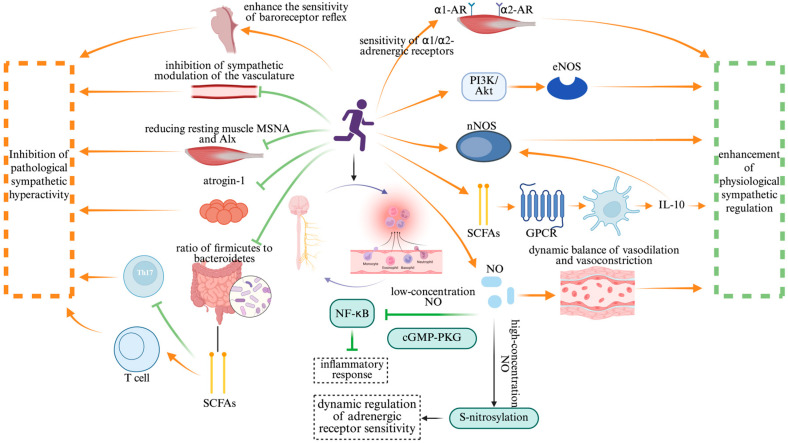
The bidirectional regulatory effect of exercise intervention. Note: Created in https://BioRender.com; 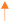: promote; 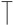: inhibit. Abbreviation: nNOS: neuronal nitric oxide synthase; eNOS: endothelial nitric oxide synthase; SCFAs: short-chain fatty acids; GPCR: G-protein-coupled receptor; NO: nitric oxide; cGMP-PKG: cyclic guanosine monophosphate–protein kinase G; α1-AR: alpha-1 adrenergic receptor; α2-AR: alpha-2 adrenergic receptor; PI3K-AKT: phosphatidylinositol-3-kinase/protein kinase B; NF-κB: nuclear factor-kappa B; S-nitrosylation: S-nitrosylation of proteins.

Exercise intervention plays a crucial role in modulating the ANS–immune axis, ensuring physiological homeostasis through the dynamic balance of sympathetic and parasympathetic activity (Figure 4). The regulatory mechanisms involve intricate molecular signaling pathways and immune modulation, contributing to overall health maintenance. This mechanism exhibits remarkable bidirectionality: on one hand, exercise mitigates pathological sympathetic overactivation, thereby suppressing excessive inflammation; on the other hand, it enhances physiological sympathetic regulation, which is essential for cardiovascular homeostasis. This bidirectional response underlines the precision adaptability of exercise in maintaining systemic health.

In the context of CVD therapy, exercise has been shown to suppress sympathetic overactivity and restore immune balance. A randomized controlled trial on patients with subclinical Chagas cardiomyopathy demonstrated that exercise training significantly reduced resting muscle sympathetic nerve activity and the aortic wave reflection index. These improvements were attributed to the enhanced baroreflex sensitivity and reduced vascular sympathetic modulation [40]. Additionally, exercise enhances the expression of genes related to skeletal muscle oxidative metabolism, while downregulating the ubiquitin-proteasome system factor ubiquitin ligase atrogin-1, thereby coordinating sympathetic inhibition with skeletal muscle metabolic remodeling. This effect is further influenced by exercise-induced alterations in the gut microbiota composition, particularly a decreased Firmicutes/Bacteroidetes ratio. These microbiome shifts promote the production of SCFAs, which, in turn, facilitate regulatory T-cell (Treg) differentiation, ultimately suppressing Th17-mediated autoimmune responses [41].

Beyond its inhibitory effects, exercise also enhances physiological sympathetic regulation by improving receptor sensitivity and signal transduction efficiency. Animal studies have demonstrated that four weeks of treadmill exercise at varying intensities dose-dependently enhances adrenergic α1/α2 receptor-mediated vasoconstriction in the resting skeletal muscle. This effect involves a neuronal nitric oxide synthase (nNOS)-dependent mechanism, which strengthens the contraction-mediated “sympatholysis” [42]. In autoimmune disease models, exercise-induced gut microbiota alterations activate G-protein-coupled receptors (GPCRs) via SCFAs, promoting dendritic-cell-derived IL-10 release and upregulating the nNOS expression to mediate nitric oxide (NO)-dependent immune modulation [41]. This seemingly paradoxical effect highlights the bidirectional nature of exercise regulation: while suppressing pathological sympathetic overactivation, exercise concurrently enhances nNOS expression and NO bioavailability, thereby improving the dynamic vascular response to sympathetic stimuli during physical activity. Moreover, clinical studies further support these findings, showing that exercise improves adrenergic-receptor-mediated vasoconstriction in hypertensive patients, allowing sympathetic regulation to precisely match metabolic demands [43]. This adaptive response is closely linked to exercise-induced improvements in endothelial shear stress, which sustainably activate endothelial nitric oxide synthase (eNOS) through the PI3K/Akt pathway. This pathway modulates adrenergic receptor subtype localization and optimizes the signal transduction efficiency, reinforcing the physiological adaptability of sympathetic control.

In the bidirectional regulation of the ANS–immune system by exercise, NO serves as a critical molecular hub. Its dual role is evident in the low concentrations of NO suppressing NF-κB activation via the cyclic guanosine monophosphate–protein kinase G (cGMP-PKG) signaling pathway, thereby alleviating inflammatory responses. Meanwhile, exercise-induced NO release enhances adrenergic receptor sensitivity through S-nitrosylation, dynamically modulating sympathetic activity [44]. These findings underscore that exercise exerts a multi-level “neuro–immune–metabolic” regulatory effect, enabling the bidirectional correction of pathological sympathetic overactivation and immune imbalance. This intricate interplay highlights exercise as a precise modulator of autonomic and immune homeostasis, reinforcing its therapeutic potential in inflammatory and autonomic-dysfunction-related disorders.

Overtraining can disrupt ANS function, typically characterized by the compensatory suppression of sympathetic activity and heightened parasympathetic tone. This autonomic imbalance is closely associated with the development of exercise-induced fatigue and depressive behaviors [45]. From a metabolic perspective, overtraining enhances the oxidation of branched-chain amino acids (BCAAs), thereby increasing the competitive transport of tryptophan across the blood–brain barrier and promoting its conversion into serotonin, contributing to CNS fatigue [46,47]. Concurrently, pathologically elevated levels of ROS and the excessive release of inflammatory cytokines such as IL-6 and TNF-α trigger a state of systemic low-grade inflammation, which further exacerbates impairments in muscle energy metabolism [48]. Notably, individuals subjected to chronic excessive exercise often exhibit a marked reduction in plasma glutamine levels, which inhibits lymphocyte proliferation and antibody synthesis, ultimately compromising immune function [49]. Collectively, these molecular mechanisms underscore the critical need to accurately identify individual exercise thresholds to guide precise dose–response interventions. This is essential for optimizing exercise prescriptions while minimizing potential immunological risks.

## 4. Precision-Medicine-Driven Personalized Exercise Interventions

The rapid advancement of precision medicine has provided a scientific foundation for the personalized design of exercise interventions. The core of this approach lies in deciphering the biological mechanisms underlying individual variability and optimizing intervention strategies based on multidimensional biological data.

### 4.1. Individual Variability Analysis Based on Multi-Omics Approaches

The variability in individual responses to exercise arises from genetic polymorphisms and the complexity of metabolic regulatory networks. Genomic studies have identified the intricate relationship between genes associated with the autonomic nervous system and skeletal muscle metabolism as key determinants of exercise efficacy. For instance, the I/D polymorphism of the ACE gene has been linked to aerobic endurance performance, with individuals carrying the I allele exhibiting a greater improvement in VO2max in response to endurance training compared to those with the D allele [50]. Similarly, the R577X polymorphism of the ACTN3 gene, which encodes α-actinin-3, is associated with power-based exercise performance. Individuals with the XX genotype, lacking α-actinin-3 expression, display a weaker muscle hypertrophic response to resistance training [10]. Moreover, skeletal muscle peroxisome-proliferator-activated receptor gamma coactivator 1-alpha (PGC-1α) gene expression plays a pivotal role in mitochondrial biogenesis and energy metabolism. Individuals carrying the GG genotype of PGC-1α rs8192678 exhibit more significant improvements in maximal oxygen uptake following endurance training, while those with the AA genotype demonstrate enhanced muscle fiber adaptability in response to resistance training [11]. Additionally, the C allele of the peroxisome-proliferator-activated receptor delta (PPARδ) rs2016520 gene enhances the fatty acid oxidation capacity, significantly improving the fat utilization efficiency after endurance training. Furthermore, transgenic mice with the skeletal-muscle-specific overexpression of PGC-1α have been shown to upregulate kynurenine aminotransferases (KATs), thereby reducing plasma kynurenine (KYN) levels and mitigating stress-induced depressive behavior [51]. This suggests that gene expression profiles may serve as biomarkers for predicting the anti-inflammatory effects of exercise, highlighting genotyping as a crucial factor in tailoring exercise regimens for individuals.

Metabolomics analyzes individual responses to exercise interventions from the perspective of dynamic metabolism. The post-exercise elevation of plasma BCAA is associated with enhanced muscle protein synthesis; however, the efficiency of BCAA metabolism is regulated by gene–environment interactions. Notably, individuals carrying specific variants of the branched chain amino acid transaminase 2 gene exhibit accelerated BCAA clearance rates following high-intensity interval training (HIIT), leading to a significantly improved muscle repair capacity [51]. The exercise-induced reprogramming of the KYN metabolism also demonstrates substantial inter-individual variability. Individuals with a high kynurenine aminotransferase (KAT) activity show an increase in the plasma kynurenic acid (KYNA)/KYN ratio from 0.08 to 0.15 after HIIT, which is associated with a significant reduction in depression risk. In contrast, individuals with a low KAT activity can activate alternative anti-inflammatory pathways through moderate-intensity continuous exercise (MICE) [52]. Furthermore, individuals carrying the PPARδ C allele, who are metabolically predisposed to enhanced lipid oxidation, exhibit significantly improved fatty acid oxidation efficiency during HIIT. Meanwhile, those with a high glucose transporter type 4 (GLUT4) expression, characterized by a heightened glucose metabolism sensitivity, benefit from MICE in optimizing insulin sensitivity [12]. These gene–metabolism interactions provide molecular targets for the development of precision exercise prescriptions.

The integration of multi-omics data provides a molecular basis for the personalized design of exercise prescriptions. Individuals carrying the synaptotagmin 10 (SYT10) risk allele and exhibiting low levels of high-density lipoprotein cholesterol (HDL-C) have a diminished capacity for parasympathetic reactivation. For these individuals, prolonged low-intensity exercise is recommended to mitigate the risk of delayed heart rate recovery [53]. For patients with resistant hypertension, those with the ADRA1A rs544215 TT genotype demonstrate a significantly greater reduction in office blood pressure in response to HIIT compared to MICE. In contrast, individuals carrying the CC genotype require resistance training in conjunction with aerobic exercise to improve endothelial function [54]. Machine-learning models enable the quantification of gene–metabolism–phenotype interaction networks. By integrating genetic risk scores such as ACE I/D and ACTN3 R577X, metabolic markers such as BCAAs/KYN, and clinical parameters such as the heart rate and insulin sensitivity, predictive models can precisely recommend HIIT or MICE as targeted exercise interventions [55].

The optimal threshold for exercise prescription is jointly determined by genetic predisposition and individual metabolic characteristics. In terms of exercise intensity, individuals with the PGC-1α rs8192678 GG genotype exhibit a higher threshold for mitochondrial biogenesis and, thus, require HIIT at ≥85% VO2max to effectively activate the AMPK-PGC-1α signaling pathway. In contrast, carriers of the AA genotype achieve maximal metabolic benefits at a lower exercise intensity of 60–75% VO2max. Regarding exercise frequency, individuals carrying the IL-6 rs1800795 G allele, characterized by a lower sensitivity to inflammatory responses, can tolerate up to five HIIT sessions per week without adverse effects. However, individuals with the C allele should limit their HIIT sessions to three times per week to avoid the cumulative risk of muscle damage [56]. Furthermore, real-time physiological monitoring via metabolomics allows for dynamic adjustments in exercise prescriptions. For example, individuals whose plasma lactate/pyruvate ratio remains elevated beyond 24 h post-exercise may require a reduction in training intensity and an extension of recovery periods [57]. Despite these advancements, the current multi-omics integration research still faces challenges in overcoming the limitations of static analysis. For instance, circRNA DICAR has been found to inhibit cardiomyocyte apoptosis by binding to Valosin-containing protein (VCP) in diabetic cardiomyopathy, and exercise interventions can upregulate its expression to counteract metabolic dysfunction [58]. To decode such epigenetic–metabolic interaction networks dynamically, a combination of single-cell sequencing and multi-time-point metabolite profiling is required to capture transient regulatory events induced by exercise. The integration of artificial-intelligence-driven multimodal data—including genetic risk assessment, wearable device analytics, and dynamic metabolic monitoring—will facilitate the transition from generalized exercise recommendations to real-time, personalized adaptations. This paradigm shift will, ultimately, enhance precision medicine approaches for cardiovascular health interventions.

### 4.2. Optimization of Exercise Prescriptions Guided by Dynamic Biomarkers

Dynamic biomarkers serve as critical indicators that provide real-time insights into an individual’s physiological state and adaptive responses to exercise, offering a feasible technological pathway for optimizing exercise prescriptions from a precision medicine perspective. With rapid advancements in wearable biosensor technology, artificial intelligence algorithms, and multimodal data integration platforms, the application of a dynamic-biomarker-guided exercise prescription is transitioning from theoretical exploration to clinical practice. The core principle lies in establishing a closed-loop system of “monitoring–analysis–feedback–adjustment,” allowing for real-time modifications to exercise prescriptions based on individual physiological responses.

The identification of dynamic biomarkers requires physiological specificity, assessability, and clinical relevance. Cardiovascular-risk-associated markers such as HRV and baroreflex sensitivity provide insights into autonomic nervous system function, while inflammatory factors, such as IL-6 and C-reactive protein and metabolic biomarkers like lactate threshold and blood glucose fluctuations, reflect exercise-induced immunometabolic regulation. Recent studies have demonstrated the feasibility of a real-time physiological assessment through the integration of electrocardiography (ECG), blood oxygen, and respiratory sensors for continuous cardiovascular monitoring in infants [59]. Furthermore, the incorporation of smart textiles and epidermal electronic devices enables the continuous tracking of changes in exercise intensity, capturing key physiological parameters such as autonomic nervous system tension, microcirculatory blood perfusion, and oxidative stress levels. These advancements allow for the real-time assessment of autonomic and immune system function, facilitating precision-driven exercise interventions.

The key advantage of dynamic biomarkers lies in their temporal resolution, necessitating machine-learning algorithms to decode their nonlinear relationships with exercise dosage (intensity, frequency, and duration). Recent studies have employed AI-driven exercise prescription systems for rehabilitation in lung cancer patients, utilizing cardiopulmonary exercise testing data and real-time heart rate feedback to dynamically adjust aerobic and resistance training protocols. This approach has significantly improved the rate of patients meeting their effective exercise duration targets [60]. Additionally, activation functions derived from ECG feature extraction can model ventricular elasticity changes, enabling the development of cardiovascular hemodynamic models that integrate blood flow simulation to optimize resistance training strategies dynamically [61]. These cases highlight the advantages of wearable devices and adaptive algorithms. On the one hand, recurrent neural networks or reinforcement-learning models can predict an individual’s optimal exercise load. On the other hand, cloud-based platforms facilitate collaborative decision-making between clinicians and AI algorithms, ensuring both the efficacy and safety of clinical interventions.

The effectiveness of dynamic-biomarker-guided exercise prescriptions must be validated through prospective clinical trials. Current studies often adopt adaptive trial designs, adjusting exercise protocols in response to real-time biomarker fluctuations. For instance, in heart failure patients, the continuous monitoring of N-terminal pro-B-type natriuretic peptide (NT-proBNP) and HRV has been used to assess exercise-induced cardiac remodeling risks. Based on these dynamic assessments, exercise intensity is adjusted to prevent potential injury [62]. Furthermore, advancements in multi-omics technologies have enhanced the precision of dynamic exercise prescriptions. Genomic markers such as ACE I/D have been utilized to predict individual vascular adaptations to endurance training, while metabolic biomarkers enable the identification of inter-individual differences in post-exercise lipid metabolism, providing a molecular basis for personalized exercise interventions [50,51,53]. Despite these promising developments, the clinical application of dynamic biomarkers faces significant challenges. First, integrating the spatial and temporal heterogeneity of biomarkers remains a critical hurdle, requiring the development of multi-scale modeling approaches. Second, a real-time data analysis is essential for dynamically adjusting exercise prescriptions; however, the limitations in data collection, transmission delays, and measurement errors necessitate breakthroughs in iterative edge computing and 5G transmission technologies. Additionally, ethical and privacy concerns surrounding biomarker monitoring call for the establishment of standardized data anonymization protocols. Future research should explore the construction of personalized autonomic–immune cardiovascular virtual models to simulate biomarker trajectories across various exercise scenarios, enabling the pre-optimization of exercise interventions and risk prediction [63].

### 4.3. Clinical Translation: Case Studies and Challenges

Precision-medicine-driven exercise prescription interventions for cardiovascular disease management have progressively transitioned from theoretical exploration to clinical practice. The “SMART-CR/SP” study conducted in China was the first to validate the effectiveness of a WeChat-based cardiac rehabilitation model. By integrating personalized educational modules, the remote physiological monitoring of parameters such as blood pressure and step count, and real-time bidirectional feedback, this intervention significantly improved patients’ functional exercise capacity and medication adherence [64]. The success of this model can be attributed to its precise behavioral profiling and the high penetration rate of its technology platform, embodying a “patient–technology–environment” triadic precision design approach. In Germany, a “HIP-in-Würzburg” study developed a structured group-based weekly training program for heart failure patients, integrating wearable devices to monitor daily physical activity. After 12 months of intervention, patients exhibited a significant improvement in the left ventricular ejection fraction and Kansas City cardiomyopathy questionnaire scores, reflecting an enhanced quality of life [65]. This model effectively addressed the exclusion of high-risk patients from traditional cardiac rehabilitation programs by incorporating physician–patient collaborative supervision and risk stratification based on NYHA class II–III. An “Active-at-Home-HF” trial in the United Kingdom introduced a home-based intervention centered around incremental step-count goals, targeting a daily increase of 2000 steps. The program utilized telephone follow-ups for supervision and dynamic step-count adjustments, with 85% of participants completing the 12-week intervention and demonstrating a significant improvement in peak stroke volume [66]. This model’s strengths lie in its low technological barrier and flexible adaptation, making it particularly suitable for individuals with mobility limitations. However, the study exhibited a gender bias, with 90% of participants being male, and its relatively short 12-week intervention period lacked long-term follow-up data, limiting the generalizability of its conclusions. These clinical cases illustrate the growing feasibility of precision exercise prescriptions but also highlight the persistent challenges in sample diversity, study design, and long-term efficacy assessment. Addressing these limitations will be crucial in refining personalized exercise interventions for broader clinical applications.

To achieve the clinical translation of precision-medicine-driven, personalized health interventions, three core challenges must be addressed. Firstly, we must balance individual variability and intervention precision. One major limitation is the reliance on group-averaged exercise prescriptions, which fail to accommodate inter-individual differences in physiological responses [67]. For instance, in the SMART-CR/SP study conducted in China, some patients with visual or cognitive impairments were unable to operate smartphone-based interventions, while, in the Active-at-Home-HF study in the UK, participants with arthritis struggled to meet the incremental step targets. To enhance precision, multi-dimensional biomarker integration—including CPET parameters, autonomic nervous system function, and epigenetic characteristics—has been proposed to classify responders into distinct subgroups. Recent studies have also explored machine-learning algorithms to predict exercise response phenotypes, such as anaerobic threshold adaptability, providing a foundation for stratified interventions. Secondly, we must overcome the technical barriers in data integration and real-time monitoring. Despite advancements in wearable technology—such as smartwatches and bioimpedance devices capable of real-time heart rate and HRV monitoring—the standardization and integration of multi-source heterogeneous data remain significant challenges [68]. For example, in the HIP-in-Würzburg study, the temporal association between step-count data and echocardiographic parameters was not fully explored. Future research should leverage emerging edge-computing technologies to enable local data processing, reducing transmission delays, and adopt federated learning frameworks to facilitate cross-center data modeling while preserving patient privacy. Thirdly, addressing long-term adherence and behavioral sustainability. A lack of sustained adherence mechanisms remains a critical barrier to the long-term efficacy of exercise interventions [64]. Many intervention benefits diminish after program completion; for instance, in SMART-CR/SP, patients’ step-count improvements declined six months post-intervention, suggesting that a technology-dependent model may weaken intrinsic behavioral motivation. According to the self-determination theory, embedding intrinsic motivation strategies—such as gamified social reward systems or adaptive goal-adjustment algorithms—could enhance participant engagement and long-term compliance.

These clinical case studies underscore the potential of precision-medicine-based exercise interventions for cardiovascular disease management, yet highlight the need to move beyond a “one-size-fits-all” paradigm. Future research should integrate genomic, metabolomic, and microbiome data to develop individualized risk prediction models and establish a “monitoring–analysis–regulation” closed-loop system, where real-time biomarker feedback automatically adjusts exercise prescriptions to optimize outcomes.

## 5. Precision Exercise and Multimodal Data Integration in Cardiovascular–Immune Regulation

Exercise interventions exert intricate regulatory effects on the ANS, immune system, and cardiovascular health, forming a highly interconnected physiological network. Recent advancements in single-cell multi-omics technologies and multimodal data integration approaches have provided unprecedented opportunities to decode the synergistic mechanisms underlying these interactions, offering a new precision medicine perspective. Single-cell transcriptomics coupled with bulk metabolomics have enabled the precise characterization of metabolic reprogramming in immune subpopulations in response to exercise. Additionally, wearable sensor technology has facilitated the real-time physiological monitoring of HRV and blood pressure variability, providing dynamic insights into ANS homeostasis [9,63]. Recent studies have integrated single-cell epigenomics, plasma metabolomics, and ANS function markers to reveal that exercise-induced vagal activation regulates oxidative phosphorylation pathways in CD8+ T-cell subsets, thereby suppressing inflammatory cytokine release and alleviating atherosclerosis progression [69]. Moreover, the emergence of spatial multi-omics technologies has made it possible to map the interactions between autonomic nerve fibers and immune cells within tissue microenvironments. For instance, sympathetic nerve terminals have been shown to regulate cardiac-resident macrophage glycolytic activity via catecholamine secretion, thereby modulating the cardiac repair efficiency [70]. Decoding such metabolic reprogramming mechanisms allows federated learning systems to dynamically align patient-specific glycolytic/oxidative phosphorylation preferences, thereby optimizing exercise intensity recommendations. These findings underscore the potential of multi-omics and real-time monitoring to enhance our understanding of the systemic effects of exercise on cardiovascular health, paving the way for precision-medicine-based exercise interventions.

Despite the vast potential of multimodal data integration, its widespread application faces multiple challenges. At the technical level, significant discrepancies exist in the temporal resolution, spatial scale, and data dimensionality between different data modalities, such as single-cell multi-omics data (e.g., transcriptomics, proteomics, and metabolomics) and dynamic physiological signals (e.g., HRV and ECG). These differences make it difficult for traditional statistical models to achieve cross-modal alignment. For instance, there is a notable temporal scale discrepancy between the immediate ANS response to exercise in the range of seconds and the metabolic reprogramming of immune cells which occurs over hours. Current computational algorithms struggle to effectively capture the dynamic coupling between these processes. At the biological level, previous discussions have highlighted how autonomic neural signaling regulates the systemic immune status via the “brain–immune axis” and how the immune system, in turn, provides feedback modulation to the ANS. However, in the context of CVD, several pathways remain unclear. Network inference based on multi-omics data is highly susceptible to confounding effects from redundant associations. For example, Schafer’s ligand–receptor interaction-based approach to predicting cell communication often overlooks the paracrine effects of small metabolic molecules such as lactate and ATP, leading to inconsistencies in the interpretation of the “exercise–vagus nerve–immune” pathway. Furthermore, additional confounding factors, including individual genetic variations and gut microbiota composition, further complicate the causal inference of multimodal data integration, making it challenging to establish robust mechanistic links [70].

To overcome the existing limitations in multimodal data integration, both methodological innovation and systems biology approaches are required. From a methodological perspective, it is essential that we develop adaptive multimodal alignment algorithms and construct heterogeneous embedding models based on graph neural networks. These models can map single-cell multi-omics features and continuous physiological signals into a unified latent space, facilitating the identification of cross-modal biomarkers. For instance, such approaches could reveal coordinated changes between the fatty acid oxidation gene expression in specific CD4^+^ T-cell subsets and low-frequency power in HRV. From a systems biology standpoint, a paradigm shift is needed to develop dynamic “molecule–cell–organ” models. This requires integrating single-cell regulatory networks inferred by single-cell regulatory network inference and clustering, metabolic flux analyses, and ANS–immune coupling differential equations. By quantifying the dose–response relationship between cardiovascular immune homeostasis and exercise parameters including modality, intensity, and frequency, these models can offer valuable insights. For example, virtual clinical trials could predict the optimal threshold at which HIIT activates the cholinergic anti-inflammatory pathway to suppress monocyte TNF-α secretion. More importantly, advancing precision medicine applications is crucial. By leveraging federated learning, multimodal data from multicenter cohorts can be integrated to develop personalized exercise prescription recommendation systems. These systems could dynamically optimize intervention strategies based on an individual’s baseline immune–metabolic profile, such as the proportion of S100A9^+^ inflammatory monocytes and vagal tone. This approach marks a fundamental transition from “population-based generalization” to “individualized precision”, facilitating highly tailored exercise interventions for cardiovascular and immune health [71].

## 6. Conclusions

Exercise serves as a pivotal modulator of the autonomic–immune axis, restoring cardiovascular homeostasis by rebalancing sympathetic–vagal activity and modulating immune responses. From a precision medicine perspective, the integration of multi-omics technologies and dynamic biomarkers enables the stratification of individual exercise responsiveness, thereby guiding personalized intervention strategies. Emerging clinical applications—such as smart remote rehabilitation and AI-driven adaptive training—demonstrate the feasibility of translating these insights into practice. However, challenges persist in harmonizing multidimensional data streams, ensuring sustained patient adherence and democratizing access to advanced monitoring technologies.

To propel this field forward, future efforts must prioritize the following: (1) decoding spatiotemporal mechanisms by coupling single-cell multi-omics with real-time physiological signals to unravel the dynamic interplay between exercise, autonomic regulation, and immune modulation; (2) engineering scalable platforms that leverage edge computing and federated learning for the privacy-preserving, real-time optimization of exercise prescriptions; and (3) building predictive virtual twin models through systems biology–AI integration to simulate individualized cardiovascular responses under diverse exercise scenarios. By embracing data-driven, algorithm-enhanced frameworks, the paradigm of cardiovascular protection can evolve from empirical guidelines to precision dynamic regulation, ultimately improving patient outcomes and reducing the global burden of cardiovascular diseases.

## Data Availability

Not applicable.
